# Consensus communication strategies to improve doctor-patient relationship in paediatric severe asthma

**DOI:** 10.1186/s13052-019-0623-0

**Published:** 2019-03-04

**Authors:** Antonietta Cappuccio, on behalf of Paediatric Sound Group, Filomena Bugliaro, Silvia Maria Elena Caimmi, Valeria Caldarelli, Lucia Caminiti, Enza D’Auria, Emanuela di Palmo, Marzia Duse, Alessandro Giovanni Fiocchi, Francesco Gesualdo, Ahmad Kantar, Enrico Lombardi, Anna Lucania, Margherita Marchiani, Maria Giulia Marini, Gianluigi Marseglia, Maria Carmela Montera, Elio Massimo Novembre, Guido Pellegrini, Giorgio Piacentini, Alessandro Policreti, Francesca Santamaria, Filomena Bugliaro, Silvia Maria Elena Caimmi, Valeria Caldarelli, Lucia Caminiti, Enza D’Auria, Emanuela di Palmo, Marzia Duse, Alessandro Giovanni Fiocchi, Francesco Gesualdo, Ahmad Kantar, Enrico Lombardi, Anna Lucania, Margherita Marchiani, Maria Giulia Marini, Gianluigi Marseglia, Maria Carmela Montera, Elio Massimo Novembre, Guido Pellegrini, Giorgio Piacentini, Alessandro Policreti, Francesca Santamaria

**Affiliations:** 1 Healthcare Area, ISTUD Foundation, Via Vittor Pisani 28, 20128 Milan, Italy; 2 Patient Association Federasma e Allergie, Parma, Italy; 3 Pediatric Clinic of the University of Pavia - IRCCS Policlinico San Matteo Foundation, Pavia, Italy; 4 Pediatric Clinic, Azienda Ospedaliera Santa Maria Nuova, Reggio Emilia, Italy; 5 Department of Pediatrics, Allergy Unit, University of Messina, Messina, Italy; 6 Department of Pediatrics, V. Buzzi Children’s Hospital, University of Milan, Milan, Italy; 7 Pediatric Clinic, Ospedale S. Orsola-Malpighi, Università di Bologna, Bologna, Italy; 8 Department of Pediatrics and child Neuropsychiatry, University Sapienza, Rome, Italy; 9 Pediatric Clinic, Ospedale Pediatrico Bambino Gesù, Roma, Italy; 10 Pediatric Asthma and Cough Centre, Istituti Ospedalieri Bergamaschi University and Research Hospitals, Ponte San Pietro-Bergamo, Italy; 11 Pediatric Pulmonary Unit, “Anna Meyer” Pediatric University Hospital, Florence, Italy; 12 Pediatria ospedale dei bambini arnas civico, Palermo, Italy; 13 Patient Association Respiro Libero, Milano, Italy; 14 Department. of Medicine, Unit of Allergology and Clinic Immunology, AOU S.Giovanni di Dio e Ruggi d’Aragona, Salerno, Italy; 15 Allergic Unit, Azienda Ospedaliero- Universitaria A Meyer, Firenze, Italy; 16 U.O. of Pediatric and Neonatology, Hospital City of Sesto San Giovanni (MI), ASST Nord Milano, Milan, Italy; 17 Broncopneumologia Pediatrica AOUI, Verona, Italy; 18 Medical department, Novartis Farma, Origgio, Italy; 19 Department of Translational Medical Sciences, Federico II University, Naples, Italy

**Keywords:** Severe asthma, Paediatric, Communication, Doctor-patient relationship, Consensus

## Abstract

**Background:**

Asthma is a chronic inflammatory disease that is very common among youth worldwide. The burden of this illness is very high not only considering financial costs but also on emotional and social functioning. Guidelines and many researches recommend to develop a good communication between physicians and children/caregiver and their parents. Nevertheless, a previous Italian project showed some criticalities in paediatric severe asthma management. The consensus gathered together experts in paediatric asthma management, experts in narrative medicine and patient associations with the aim of identify simple recommendation to improve communication strategies.

**Methods:**

Participants to the consensus received the results of the project and a selection of narratives two weeks before the meeting. The meeting was structured in plenary session and in three working groups discussing respectively about communication strategies with children, adolescents and parents. The task of each working group was to identify the most effective (DO) and least effective practices (DON’ T) for 5 phases of the visit: welcome, comprehension of the context, emotions management, duration and end of the visit and endurance of the relationship.

**Results:**

Participants agreed that good relationships translate into positive outcomes and reached consensus on communication strategies to implement in the different phase of relationships.

**Conclusions:**

The future challenges identified by the participants are the dissemination of this Consensus document and the implementation of effective communication strategies to improve the management of pediatric asthma.

## Background

Asthma is a heterogeneous chronic inflammatory disease that affects airways causing respiratory symptoms including wheeze, breathlessness, chest tightness, and cough [1, 2]. Asthma is very common among youth worldwide, with approximately 7% of adolescents and 5% of children reporting symptoms of severe asthma [3]. The burden of this disease is very high and it causes numerous school absence and visits to the emergency department (ED) [4]. In the USA, Wand and colleagues have estimated that the management of paediatric asthma costs 2 billion dollars per year in direct and indirect expenses [5]. Medication costs differ across countries depending on the health system but in recent years the use of asthma drugs is globally increased [6, 7]. Nevertheless, in Italy one of the major problems in asthma management is represented by poor adherence to the therapy and to the implementation of an appropriate lifestyle [8].

Poorly controlled asthma doesn’t affect only the expenses but also lead to an increase in morbidity and mortality [9]. At the same time, a delay in the treatment of asthma undermine patients’ and caregivers’ emotional, psychological and social functioning by limiting their ability to engage in normal day activities [10, 11].

In order to improve asthma management, guidelines recommend developing partnership between healthcare providers, patients and their parents.^1^ [12] Many studies demonstrated the efficacy of health communication intervention to improve the doctor-patient/parent relationship and diminish non-adherence and access to ED. [13–16] The benefit of doctor-patient communication may extend beyond these short-term outcomes, as demonstrated on other adult chronic diseases [17–19].

Despite this evidence, children and adolescents are rarely engaged in discussion during medical visits, accounting for only 3–15% of the total medical visit interactions [20]. Indeed Carpenter and colleagues demonstrated that providers engage their patients less frequently than these children and adolescents preferred [13].

In 2016, ISTUD Foundation led a project named SOUND (the Italian abbreviation of “Writing narrative about patients with severe asthma to achieve a new effective diversification and improvement in healthcare”) to investigate doctor-patient relationship in severe asthma through the narrative medicine methodology [21]. Narrative medicine research seeks to gain insight into how a person lives with his/her illness, in an attempt to consider the many facets of the pathway of care [22–24].

The results of the SOUND project highlighted the need to improve the communication to children and adolescents with severe asthma and their parents since paediatricians recurred more frequently to judgemental words compared to providers caring adult patients and this endanger the building of mutual trust [21].

In view of what emerged from the SOUND project, it was considered that the “Consensus” was an appropriate method to identify first and then share a series of attitudes and behaviours to be adopted in daily clinical practice, in order to obtain effective relationships with paediatric patients with severe asthma and their families.

## Methods

The methodology of building consensus is a decision-making process that aims to identify what is preferable for a group through a face-to-face debate among stakeholders [25]. The consensus methodology aims to move from the logic of voting to that of deliberation, improving the decision-making process [26] through rational and emotional arguments to limit or renounce particular interests in light of the collective interest [27]. One kind of consensus conference uses a group of experts [28] who meet in an open meeting to hear evidence and try to reach a consensus on procedures to follow [26]. One of the advantages of this methodology is to strengthen the engagement of the experts involved in the decision-making process, generating greater awareness in the carrying out of activities with respect to objectives [29].

The results of the SOUND project and a selection of narratives collected within the project were sent to the members of the Paediatric SOUND group two weeks before the meeting to allow all participants to analyse the evidences and make their own opinion. The consensus meeting was performed on the 14 September 2017 and held at the headquarters of the ISTUD Foundation in order to reduce the influence provided by the location since it was new to any participant. The participants were representative of three different groups of experts:

- 11 paediatricians with strong expertise in paediatric severe asthma management (eg. presidents of Italian scientific societies, professors);
- 6 paediatricians who already participated in the SOUND project and which showed a strong disposition to communication;
- 3 representatives of patient association which are patients or parents of children and adolescents with asthma.

The researchers of ISTUD Foundation, due to their expertise on narrative medicine and on the SOUND project, had the role of facilitator of the meeting.

The first plenary session of the meeting was aimed to reaffirm the main results of the SOUND project. Subsequently, the objectives of the day and the Consensus methodology were shared with practical examples linked to work previously carried out by the ISTUD Foundation. Three working groups were therefore set up, based on the professional and roles of the participants involved to maintain wide representativeness. This promoted the mutual exchange of experience, good practice, and new ideas.

The groups worked on three specific macro-topics related to the theme of communication in the care pathway for severe asthma, respectively focusing on communication to children, communication to adolescents and communication to parents.

The task of each working group was to identify the most effective (DO) and least effective practices (DON’ T) for 5 phases of the visit identified by the ISTUD Foundation researchers: welcome, comprehension of the context, emotions management, duration and end of the visit and endurance of the relationship.

The communication phase of the diagnosis and the phases most linked to the disease were not considered in an analytical way, as the project SOUND did not reveal any particular critical points in the management of this type of communication for Italian scenarios.

During the subdivision of the working groups, each participant had a few minutes to write their own proposals of DOs and DON’Ts for each phase of the visit. Subsequently, the facilitators of the groups gathered the written ideas and the whole group discussed the ideas to arrive at a series of actions to do and not do for each phase. At the end of the group work, participants voted anonymously on the degree of consensus reached for each phase using a scale from 1 to 10, only the proposal that reached at least a degree of 8.5 where selected [26]. All the practices and ideas proposed by the individual groups were shared and discussed in plenary.

After the meeting, the results of the consensus were collected by ISTUD Foundation and shared with the Paediatric Sound Group, which had one month to review all the results and confirm, or not, their degree of consensus.

## Results

From reading the results of the SOUND project, the consensus participants agreed that the relationships and the ability to communicate empathically with patients and their families are the aspects that cross all the collected narratives. In particular, the ability to establish positive relationships affects not only people’s experiences but also the outcomes of the therapeutic plan [21].

During the course of the meeting, members from all groups actively worked together to develop proactive proposals and strategies to improve the relationship between physicians and patients with severe asthma.

Tables 1, 2, 3, 4, 5 show the reflections and results of the discussion, the consensus degree reached by the total of the proposal is 9.4 out of 10. DOs and DON’Ts were written by the participants based on their experiences in the management of severe asthma, even if some suggestions can be applicable to all chronic diseases.

**Table 1:** Proposals and strategies identified by the Pediatric Sound Group for the “Welcome” phase

	DO	DON’T
Welcome (consensus degree 9.9)	• The waiting room should consist of two spaces, one suitable for children and one for adolescents • Select educational material earmarked to parents and teenage patients to place in the waiting room. • Make sure that the child is not afraid of the doctor and the environment • Before taking care of the family, prepare for who they are and what their situation is. • During the reception phase, smile and stand up to welcome the family. In case of new patients, you should present yourself first and explain the “rules” of the clinic • Make sure that there are enough chairs for everyone, including the father, and games for young children • Get into relationship gradually, without asking immediately about asthma • Make the children understand that they should not be afraid and transform diagnostic examinations into games (e. g. Prick-test as tattoos, Spirometry as a computer game) • Try to limit the factors that disturb the visit, such as phone calls and interruptions by colleagues, and always provide an explanation to the patient and their family on the reasons for the interruption.	• Don’t force the children to sit down but let them move freely inside the room. It could be useful in order to understand their behavior. • Do not use technical language • In the management of adolescents, it is advisable not to intervene with educational advice addressed to parents in the presence of the child. On the other hand, it is appropriate to share with parents the approach to the most restless children. • Do not schedule two first visits one after the other; families that come back for control can reassure and support new families.

**Table 2:** Proposals and strategies identified by the Pediatric Sound Group for the “Comprehension of the context” phase

	DO	DON’T
Comprehension of the context (consensus degree 9.3)	• First of all, ask “What is your reason for this visit?”; while if it were a check-up, the question could be replaced by: “How have you been since our last visit?”. Secondarily, try to ask questions about nutrition, physical activity, relation with siblings and school progress, trying to understand also the reasons behind the answers. • Listen to the opinion of the child, in addition to that of the parents: it allows a direct dialogue and makes them feel involved. This practice is particularly useful when you have the impression that something is hiding behind the words of the family. • Invite the child to draw, for example, a child in the rain or a family. Reading and interpreting the drawing can help to understand how the child feels. This practice could also be used with parents while performing examinations on the child, especially if they are distressing (recalling the positive effects of Art-Therapy).	• For adolescent patients, do not ask the parents if the teenager can answer alone. • Don’t show disappointment, but declare it calmly • Do not directly ask for parents’ profession or judge the parental style • Do not impose anything or explain the same thing several times to “convince”.

**Table 3:** Proposals and strategies identified by the Pediatric Sound Group for the “Emotions management” phase

	DO	DON’T
Emotions management (consensus 9.4)	• If the patient and their family have been treated by other physicians before, try to solve negative aspects of their experiences, without diminishing the work of others. • If the child or his family is getting angry, continue with the physical visit and interrupt the speech to recover the calm. • If the family share a problem (as divorce or grief) with you, comfort them and explain that their circumstance is common among patients and that this will not affect the relationship • Adolescent patients must feel that they are the protagonists and that their experience is acknowledged • If the patient (or family) smokes or is not adhering to the therapies, it is necessary to raise awareness of the severity of these actions with an understanding attitude. • Ask for explanations of the behavior or if something is not clear, making explicit to the patient and family what hinders the relationships • Choose the words to be used according to the interlocutor and respond to fears by presenting scientific data in an appropriate language • Agree on actions with patients/parents • Admit your emotional difficulty with colleagues by asking for support from a departmental psychologist or an evolutionary psychologist if you feel the need for it	• During the visit, avoid judging, imposing and simplifying. • Never forbid the child anything (e. g. plush toys, pets, sports) but find a planned compromise with the child and their clinical situation. • If parents belittle a problem, don’t load them with excessive emotional weight, but take them gently and over time to understand the situation. • If the family or child makes points that are very important to them, do not underestimate their expectations. • Do not respond to provocations and at the same time do not place yourself above the interlocutor, emphasizing the hierarchy.

**Table 4:** Proposals and strategies identified by the Pediatric Sound Group for the “Visit end” phase

	DO	DON’T
Visit end (consensus 9.9)	• Use the indicative verbal time and the plural to make the family understand that they can count on the entire care team. • Explain in detail the therapy and behavior to be adopted at home and school, agreeing with the adolescent or family to manage the disease at home. • Before greeting the child and their family, schedule the follow-up and provide appropriate and clear therapeutic recommendations on a separate sheet • Reassure the family and make them feel welcome by providing all the contact details of the clinic and the e-mail address • Ask the child if he/she wants to go back to the next control and if he/she has been good give him/her a prize (e. g. candy, courage certificate) • Ask the family to keep a diary of acute attacks	• Do not adopt a sufficient attitude nor assume that patients and parents already know things. • Do not impose your decisions, but do not seem undecided or hesitant using the conditional verb • Prevent other doctors from taking over during the visit or delegating to other health professionals the task of explaining the pharmacological treatment. • If the visit has taken place in a freelance profession, do not oblige parents to return.

**Table 5:** Proposals and strategies identified by the Pediatric Sound Group for the “Endurance of the relationship” phase

	DO	DON’T
Endurance of the relationship (consensus degree 8.7)	• Deliver all the contact details of the clinic to the family, always remembering that in case of emergency the first thing to do is to contact the emergency room • Propose the creation of an ad hoc application to manage relationships • Ask parents and patients to keep a daily symptom log • If the patient does not appear at the check-up and does not answer by telephone, contact the family pediatrician or the general practitioner • Invite parents and the patient to write down what it means for them to live with severe asthma and deliver the narrations to the next follow-up	• Do not follow patients or parents on social networks • Do not act as an individual but always refer to and rely on the entire care team

In particular, all participants agreed to maintain a warm and reassuring attitude towards the children by trying to involve them in all phases, while adolescent patients should be treated as adults by directing the interest of the examination to them and not to their parents. Other recurring themes are the use of open questions and, especially in the early stages of the visit, not strictly related to asthma management, and the ability to contain negative emotions.

One important challenge identified by the children communication group is the prohibition topic: participants shared their experiences and they unanimously decided that it is important to never forbid the child anything (e. g. plush toys, pets, sports) but find a planned compromise with the child and their clinical situation. During the plenary session, all participants agreed that these should be the correct behaviour, since prohibitions can cause trauma in patients’ experience and negatively affect the relationship.

Some topics widely discussed within the groups have been the management of interruptions to visits by other doctors or phone calls and the possibility of sharing one’s mobile phone number with the patient’s parents. Initially, some doctors proposed to completely avoid answering the telephone and others that this was impossible in the daily management of the visits; at the end of the day the participants agreed that interruptions should be minimized and the visiting families should be reassured that they will benefit from the same availability.

The lowest degree of consensus was reached when discussing how to maintain relationships between the visits (Table 5). Paediatricians, who initially proposed to share their personal mobile phone number with families to manage the relationship remotely, through instant messaging applications (e.g. Whatsapp), after the group work, did take a step backwards. This because giving medical advice through this means of communication is not regulated and supported by law, thus all patients would refer only to one doctor and not to the entire care team. Conversely, the idea of creating an app for managing online monitoring of patients with severe asthma had been advocated by all participants.

In conclusion, the future challenges identified by the Pediatric SOUND Group are the dissemination of these suggestions and the implementation of classes addressed to pediatricians to improve communication and of effective technologies to maintain long-distance relationships.

## Discussion

The Pediatric SOUND Group agreed that good relations translate into positive outcome on patients’ quality of life, therefore pursuing them becomes a needed professional competence, as stated by other Italian clinicians [30]. The main result of this consensus was to create a simple and practical list of recommendations on how to improve communication with paediatric patients with severe asthma and their parents. In literature there are many evidences on how collaborative dialogue positively associate with parental satisfaction, adherence and the creation of effective relationships [31, 32], but a pragmatic list of advice was lacking to help and support clinicians.

All participants agreed firstly to maintain a warm and reassuring attitude towards the children and secondly to engage them in all phases; for adolescent patients recommendation was to address them as adults by directing the interest of the examination to them and not to their parents. This advice is supported by the studies of Giambra and colleagues [33] who demonstrated that provider dominance of communication may impair relationships during clinic visits for children with chronic conditions. Moreover, they suggested adopting narrative medicine techniques [23, 24] in every pediatric ward: thanks to reading the narratives of patients and their parents the physicians could collect useful information for the management and treatment of people living with asthma. This practice is already supported by evidences in other countries [34] and on other pathologies [35] and the broader understanding of illness in the social context of patients’ lives can improve outcomes and patient satisfaction [23].

Regarding the asthma management, the group agreed to the necessity for avoiding prohibitions (as having a pet or playing with plush toys) while focusing on the education of both patients and families to empower them to handle the allergen cause with a vigilant attitude. The group based this disruptive recommendation not only on their clinical experiences but also on many evidences that prohibitions could have a traumatic effect during childhood and in literature findings are already there regarding childhood obesity management [36, 37].

The main criticalities emerged from this consensus conference were related to the management of communication. During the visit, the first problem is due to interruptions caused by phone calls by other patients; the group agreed that, even if avoiding them desirable, visit interruptions are inevitable explaining the reasons to patients and families, but pediatricians must limit that. Regarding the communication in the long-distance relationship, the unresolved issue is linked to the use of Mobile Messaging Apps and therefore sharing the private phone number. Using WhatsApp to communicate with patients, in particular with chronic patients, is becoming quite a routine not only in Italy but worldwide [38]. Recent studies demonstrated that anxiety is frequent in mothers of children with asthma [39] and this can lead to a pathological use of online communication applications [40]. All members agreed that giving medical advice through mobile messaging apps, or even by SMS, is risky and that for real emergencies patients and their parents should go to ED or refer to other colleagues on call. On the other hand, the use of this communication method is part of modern life [41] and quite expected by patients and in particular by parents who feel reassured when they know they can count on the presence of a specialist 24/7. The urge of regulation on the use of web and instant messaging to communicate with patients is perceived not only in the management of severe asthma, but it is becoming a frequent topic nowadays in healthcare [42]. The Pediatric SOUND Group, in the end, conveyed that one possible solution is the implementation of a dedicated app for managing the long-term relationship between patients and their families and the whole care team.

One limitation of this consensus was the lack of involvement of children and adolescents to the meeting; this is due to the chosen methodology, which involves the exchange of ideas among a group of experts. In order to overcome the absence of this point of view, members of the patients’ association were invited to participate as experts in patients’ and parents’ perception. Another limitation was the participation of only specialist paediatricians: in Italy routine check-ups are mainly followed by paediatricians and in our healthcare system the involvement of other professional providers is limited, with no respiratory nurses as in other countries like UK. Nevertheless, it would be interesting to involve both patient and other healthcare professionals, as nurses or physiotherapists, in future activities on this topic.

Despite the limitations, this consensus provides strong suggestions that can be used to develop communication interventions in paediatric asthma management.

## Conclusion

The aim of this Consensus conference was to identify first and then share a series of attitudes and behaviours to be adopted in daily clinical practice, to obtain effective interactions with paediatric patients with severe asthma and their families. Overall, in agreement with previous studies, the communication between clinicians and paediatric patients with severe asthma and their parents was lacking [13, 20] with detrimental effect on the management of the illness [21]. At the same time effective communication, which is a mix of empathy and structured method [32, 33], improve the doctor-patient/parent relationship and diminish non-adherence and access to ED [13–16].

This document offers a practical guideline that may help paediatrician optimise the relationships with both patients and parents. In conclusion, future challenges are the dissemination of these suggestions to all pediatricians through education and training on soft skills. The implementation of this advice into the life-long learning process of paediatricians will help to ensure a proper education not only on the pathways of the disease but also on listening and communication to patients and their parents.

## Abbreviations

ED: Emergency December
SMS: Short Message Service
SOUND: the Italian abbreviation of “Writing narrative about patients with severe asthma to achieve a new effective diversification and improvement in healthcare”
UK: United Kingdom

## Footnotes

1 We use parents to refer to any primary caregiver
